# Fuel in Its Sanitary Applications
†Chemical Technology; or, Chemistry in its Applications to the Arts and Manufactures. By Dr. E. Ronalds and Dr. Thomas Richardson. Vol I, Parts 1 and 2. 8vo. London: Bailliere.


**Published:** 1855-12

**Authors:** 


### FUEL IN ITS SANITARY APPLICATIONS.†

THE two volumes of Drs. Richardson and Ronalds, on Fuel and its Applications, are without hesitation the completest, the most practical, and the most scientific works on this subject which we ever remember to have met with, either in English or foreign literature. To enter into a critical argument on a work of this character would be a useless, if not an impossible task. It is composed of no mere theories, but of choice scientific facts, ex-

---

† Chemical Technology; or, Chemistry in its Applications to the Arts and Manufactures. By Dr. E. RONALDS and Dr. THOMAS RICHARDSON. Vol I, Parts 1 and 2. 8vo. London: Bailliere.

perimental inquiries, and of general information bearing on the history and practical utility of each of the topics brought under consideration. In the course of the two volumes, there are many valuable remarks bearing on sanitary points. To some of these we shall now refer, following the authors closely, and with as little comment as possible.

*Historic Note on Fuel.*—The use of coal in England is a result of the scarcity of wood, and is of comparative recent date. King Henry III, in 1239, granted permission to the good men of Newcastle to dig coals; but this substance came into general use in London only about two hundred and fifty years ago; and at that period two ships were sufficient to carry on the trade. Proclamations in the reigns of Edward I and Elizabeth, forbad the use of stone-coal in London during the sitting of Parliament, lest the health of the knights of the shire should suffer during their residence in the metropolis. And, about two hundred years since, the citizens of London petitioned against two nuisances; these were—"Newcastle coals, on account of their stench, etc.; and hops, in regard they would spoyle the taste of drinck, and endanger the people."

*Application of Fuel to Domestic Purposes.*—A dwelling requires to be kept at a temperature ranging between 56° and 70° F. This could easily be accomplished with small cost, were it not that many circumstances tend to withdraw the heated air and diminish its temperature. First, the walls, doors, windows, etc., absorb the heat and evolve it externally; and the warm air of the room being specifically lighter than the external atmosphere, cold air will enter at every crevice below, while the heated air escapes at the upper parts of the room. It is also absolutely necessary to health, that the air which has once been respired should be replaced by fresh air, which must generally be cold. It has been calculated that five or six times the heat necessary to warm a room is lost by these causes.

*Mode of Heating.*—The open fireplace is the most ancient mode of warming dwellings, and its use prevails both in France and England. The open fireplace heats by radiation; and although much heat is thus lost, its comforts and advantages are so numerous, that it is not likely soon to lose caste. It produces in the simplest manner a thorough circulation of air. This result is not obtained in rooms heated by stoves, and supplied with air from without; and the air in such rooms therefore quickly becomes vitiated, especially if many persons are present, to the great detriment of their health.

Stoves are much used in many places; and although some are good, the complaint frequently is made that they cause headaches and coughs. Probably this arises from the air acquiring a degree of heat without a corresponding amount of moisture; thereby exerting a drying influence upon the skin and lungs. Water placed upon the stove partially remedies this evil.

*Effects of Respiration on the Atmosphere of Rooms.*—"The respiration of an individual has been ascertained to vitiate nearly 12 cubic feet of air per hour. The water exhaled by the lungs and skin amount to 589 grains (·084 lb.) in the same time; and supposing the air to be at a temperature of 59° F., and to take up about half of the quantity of moisture it is capable of sustaining at that temperature, which is about the average quantity it does take up, there will be required, in round numbers, per individual per hour, 212 cubic feet of air, to remove all the exhalations from the skin and lungs."

*Lights.*—"The substances employed for giving light require oxygen from the air in proportion to the amount consumed; and if one-third of the oxygen contained in the air be the quantity abstracted by the combustion, candles of six to the pound will each require about 10 cubic feet, wax candles about the same quantity, and oil or lamps with large burners, about 40 cubic feet per hour.

"One pound of peat requires for complete combustion from 70 to 134 cubic feet of air, at 66·2° F.; 1 lb. of average coal requires 228 cubic feet; and the same quantity of coke requires 194 to 250 cubic feet of air at that temperature."

*Lighting by Gas, Candles, and Lamps.*—The following extract, founded upon a comparison by Schubarth, of wax and stearine candles, is worthy of perusal: "It appears that, with reference to the hourly consumption, it is not unimportant whether four, six, or eight candles make up the pound. Of the last, the smallest quantity is consumed, more of the sixes, and most of the fours. While 1 lb. of wax candles, eight to the pound, burn 62 hours, 1 lb. of sixes burn on an average only 55 hours, and 1 lb. of fours only 48 hours. In the same order, 1 lb. of stearine candles will burn 57, 49, and 45 hours. The intensity of the light also varies, being greatest in candles eight to the pound, less in sixes, and least in fours, in about the proportion of 15 to 12 and 10." From a table shewing the relative cost of light from different sources, the remarkable fact is brought out—that those means of illumination which, from their supposed cheapness, are exclusively used by the poor, and partly by the rich, are those which cost most in producing light of a certain degree of brilliancy. The same amount of light costs nearly twice as much when produced from tallow candles as when obtained from Carcel's lamp; from the kitchen lamp it costs nearly three times, and from the lamp with the flat wick more than twice as much.

*Gas in Dwellings.*—Gas, as a means of lighting houses, was at first open to many serious objections. It contained many impurities; the temperature of the rooms was greatly raised; and a large quantity of carbonic acid was produced. For an equal amount of light, candles or oil lamps produce more carbonic acid than gas; yet, in consequence of the greater light which was always expected from gas, a larger amount of this offensive compound was found. "For every cubic foot of gas burnt, rather more than a cubic foot of carbonic acid is produced. A pound of London gas contains, on an average, 0·3 of hydrogen and 0·7 of carbon: it produces, when burnt, 2·7 of water and 2·56 of carbonic acid gas, and consumes 4·26 cubic feet of oxygen, which is the quantity contained in 19·3 cubic feet of air. It is thus obvious that the air of a close chamber must soon be vitiated by the combustion of gas, and that the consequences of breathing such an impure atmosphere must soon be felt by the consumer. The water evolved at the same time, in the form of steam, was generally charged with sulphur in some form or other, which, condensing on books, furniture, etc., caused much damage." The books of the Athenæum club library were said to have suffered serious injury from this cause. Now, however, most of these objections to the use of gas are partially, and in most cases wholly, removed. The consumption of coals in this metropolis during 1854, for the production of gas, exceeded 500,000 tons, which would yield about 4,500,000,000 cubic feet, and realize by its sale nearly 1,000,000*l.* "For lighting London and its suburbs with gas, there are eighteen public works, belonging to twelve companies, employing a capital, sunk in works, pipes, tanks, gasholders and apparatus, of 2,800,000*l.*, producing a yearly revenue of 450,000*l.* The number of private burners, supplied to about 40,000 customers, is 134,300; while there are 30,400 street lights, about 2,650 being in the city of London: 380 lamplighters are employed, and about 2500 persons connected with the works. There are 176 gas-holders, capable of storing 5,500,000,000 cubic feet of gas. The quantity of coal consumed in the shortest day, amounts to 890 tons in the twenty-four hours. The quantity of gas consumed in the longest night is about 7,120,000 cubic feet." (These statements apply to the year 1852.)

*Spontaneous Evolution of Gas.*—The evolution of inflammable gas has long been observed in some localities near the Caspian Sea, where they accompany the naphtha springs. Many celebrated springs, as those of Aachen, Neundorf, Harrogate, etc., evolve an inflammable gas, chiefly carburetted hydrogen. The same gas is frequently met with in salt mines; in some of them in such abundance as to be available for the purpose of illumination; and the Chinese frequently use these jets in the evaporation of the salt liquid. Several jets also occur in North-Eastern India, which the Hindoos having enclosed, regard with great veneration.

*Fire-damp in Coal Mines.*— In working the lower strata of some coal mines, large quantities of inflammable gas are frequently liberated, at times suddenly, and with such force as to dislodge many tons of coal; while, on other occasions, it issues in a continuous current, with a hissing noise. At Bedlay, near Glasgow, a stream of the gas was kept burning five weeks; and at the Wallsend Colliery, as much as ninety-five cubic feet per minute was discharged. The gas from this jet was conveyed in pipes, and served to light the town for years; and also, for a short period, the railway station. It is supposed possible that the gas, chiefly light carburetted hydrogen, may exist in mines in the liquid form, from the immense pressure of the superincumbent strata. The removal of the coal liberates the gas, which, mixing with atmospheric air in certain proportions, becomes dangerously explosive.

Davy found the most explosive mixture to consist of one volume of the gas to eight volumes of air. Although an explosion always occurred when the proportion of common air ranged between seven and fourteen volumes to one volume of the gas. Hence we see the extreme peril with which the miner followed his avocation, and the necessity for an invention which would give light without permitting an explosion of the gas. This was happily effected in the "Davy-lamp", and also in the "Geordey", as the lamp of Stevenson is called. The "Davy", consists of an oil lamp, with a chimney or cylinder of gauze wire, containing from seven hundred to a thousand meshes to the square inch, and is about six inches long, with one and one-third of an inch in diameter. When this lamp is carried into an explosive mixture of carburetted hydrogen no explosion occurs. The gas burns around the wick within the lamp, but does not travel outwards so as to kindle the firedamp without. The light is in fact put out in its endeavour to pass through the wire gauze, which conducts away so much of the heat, that the temperature of the flame falls below the white heat necessary for kindling firedamp. Some tables, shewing the good results of the use of the Davy, are appended. They shew that in the period from 1817 to 1825 (before the introduction of the Davy), out of a mean number of 2079 miners employed, the annual loss of life was 14·875, or one in 142; while in the period from 1825 to 1831, out of a mean number of 2928 miners employed, the annual loss was 8·571, or one in 345. From 12,000 to 15,000 safety lamps are in daily use in Durham and Northumberland, nine-tenths of which are Davy lamps; and although accidents have happened with them, either from carelessness or over-confidence, we have the experience of Mr. J. Taylor to prove that, during forty years in the north of England, no authenticated instance of explosion has arisen from the use of the unimpaired Davy lamps.

It also appears from these tables that the average loss of life per year in the coal-mines of Durham, Northumberland, and Cumberland, since the commencement of the Act for the inspection of mines, was 155 from all causes. The number of persons employed underground was estimated at 33,000, which shews the annual loss of life to be one in 215.

More lives are lost in Great Britain, in proportion to the numbers employed in coal mines, than on the continent; but comparing the loss of life with the quantity of coals raised, which is the true measure of the underground risk, the British miner works in far greater security than his brethren of the continent. Thus, from the tables—

| | | | | | |
|---|---|---|---|---|---|
| In Durham and Northumberland, | 1 | life is lost | for every | 84,000 | tons raised |
| In Great Britain . . . | 1 | „ | „ | 43,000 | „ |
| In Belgium . . . . | 1 | „ | „ | 34,000 | „ |

At St. Etienne, something more than in Belgium. In Germany the average is rather higher than in Belgium."

*Heating and Ventilation of the Houses of Parliament, etc.*—"For churches, theatres, and indeed all rooms where large numbers of persons are constantly congregated together, a thorough supply of pure, fresh, but at the same time genial air, is of the greatest consequence; although, until lately, it has had little attention. Our churches are for the most part filled with roasted or over-heated air: the upper parts of our theatres cannot be endured by persons of delicate constitutions; and even the Houses of Parliament have formed no exception to the general rule of imperfect ventilation. The inconveniences attending the want of proper ventilation are, however, beginning to excite attention; and although there is a diversity of opinion as to the quantity of air required by a number of persons during a given time, there can be no doubt that the error had better be an excess of fresh air, in preference to a deficiency."

"In the House of Commons, which has been warmed and ventilated under the superintendence, and according to the plans of Dr. Reid, the air is supplied from Old Palace Yard to the basement of the building; passing, first, through a fibrous veil 42 feet long by 18 feet 6 inches deep, for the exclusion of visible soot: it arrives by the heating apparatus, consisting of large chambers intersected by steam pipes, and proceeds from thence to other chambers, where it can be mixed with cold air and brought to any required temperature. The floor of the house is double, and the space below the floor can be connected by means of valves with the hot air chamber. The floor is perforated by a great number of apertures, and these are covered with hair cloth, so that the hot air, in escaping from the floor into the body of the house is well diffused, and no perceptible current is experienced. Having performed its functions, the vitiated air ascends to the ceiling, which is also double and perforated in the same manner as the floor, whence it is carried off by the draught created by a powerful fire under a chimney shaft erected in another part of the building.

"The plan adopted by Mr. Barry for warming and ventilating the House of Peers, the royal antechamber, and the public lobby, differs from that just described, both as respects the admission of air and its removal. The floors of the rooms are not perforated, and are heated, in the first instance, simply by the passage of hot air below them. The hot air then escapes by passages along the exernal sides of the rooms to the ceiling, which is divided into two compartments, the one for the admission of the warm air, entering at the side, and the other for the exit of the vitiated air. The warm air after passing below the floor to the roof becomes somewhat cooled, so that its temperature on entering the ceiling is a few degrees lower than that actually present in the room; it consequently descends to the level at which it is at once heated again, deteriorated by combustion, respiration, etc.; rises through the centre of the room, passing through the ceiling to a foul air chamber above, whence it is conducted to a chimney, and carried off by the peculiar motive power first suggested to Dr. Richardson by the production of draught. This power consists of a jet of steam, which, when produced under a pressure of 32 lbs. to the square inch, is capable of setting 217 times its bulk in motion: 10,000 cubic feet of air are thus sent gradually through the three apartments per minute; no draught of any kind is perceptible, and no inconvenience is experienced from dust or other solid particles being carried mechanically forward by the air, as is said to be the case when the air enters from the floor."

*Heating and Ventilation of the Newcastle Infirmary.* The committee of this institution, after much consideration, decided upon the open fire for the purpose of heating, and windows for ventilation. This plan was adopted as being more cheerful, and under better control. "The wards are double, each of them 106 feet by 23 feet, and 13 feet 6 inches in height. They are divided by a wall, in which are the open fireplaces, windows, ventilators, etc. This wall is perforated with circular openings, to allow a free communication for the air from window to window, which can be regulated according to the direction of the wind.

"The admission of cold air into buildings of this nature requires great care, as the slightest draught upon the patients would be productive of the worst consequences. It should be distributed as much as possible over the whole room. This effect is attained by forming each window into a kind of louvre. When open, the sash is at an angle of inclination, causing the cold air to enter above the heads of the patients, no beds being placed immediately under the windows.

"Cold air is also admitted to the centre of the rooms at a sufficient distance from the beds to produce no inconvenience. The outside walls are built, having an air vent three inches wide, communicating with the atmosphere by air-holes at the top and bottom. A current of air is thus established which prevents the deposition of moisture on the walls. From this vent the cold air is conveyed by an air channel, formed along the ceiling by the bearers or beams which carry the floor, and admitted near the centre of the room, where there is a valve, which can be closed at pleasure.

"The contaminated air is removed from the several wards by exhaustion on a very simple plan. The fire-places are placed back to back, having a malleable iron air chamber between them, protected from the action of the fire by fine clay lining. It is perforated at the top and bottom, to allow the atmosphere, which is supplied to it from the room below, to become heated and pass off by the ventilating flue. Thus the heat of the fire in the ward above is made to ventilate the one below. The uppermost ward is ventilated on the same plan, although, there is of course no fire place above it; the ventilating flue receiving additional heat at each story, becomes sufficiently warmed to draw the vitiated air from the uppermost. The power of the ventilating flue is increased by burning a jet of gas on either side of the iron chamber, which jets are intended to assist the ventilation in summer, or when the different fires are not burning, as well as in lighting the wards."

With these useful analyses of some parts of the volumes before us, we recommend their perusal to all our readers. It would be unfair, however, to the publisher, Mr. Baillière, to close the subject without a word of strong commendation on the admirable style in which the volumes are got up and illustrated.

---

